# Lipid-lowering agents for dyslipidemia in patients who were infected with HIV in Taoyuan, Taiwan

**DOI:** 10.7448/IAS.17.4.19556

**Published:** 2014-11-02

**Authors:** Shu-Hsing Cheng, Chien-Yu Cheng, Na-Lee Sun

**Affiliations:** 1 Department of Infectious Diseases, Taoyuan General Hospital, Taoyuan, Taiwan; 2 Comprehensive AIDS Care Center, Taoyuan General Hospital, Taoyuan, Taiwan

## Abstract

**Introduction:**

There is no doubt that highly active antiretroviral therapy (ART) has decreased the total mortality of HIV-infected populations drastically, and HIV has become a chronic and manageable condition. However, complications after long-term treatment of ART tarnished the great efforts. We aimed to study the effects of add-on lipid-lowering agents on ART for patients who developed hyperlipidemia after HIV treatment. The risk factors for failure to normalize lipid profile were analyzed.

**Materials and Methods:**

HIV-infected patients who visited outpatient clinics of Taoyuan General Hospital between July 2013 and January 2014 were retrospectively reviewed. Subjects who needed the management of dyslipidemia were enrolled. Total cholesterol (TC), triglyceride (TG), high-density lipoprotein (HDL) and low-density lipoprotein (LDL) were regularly followed up for at least 6 months. ART modification and add-on lipid-lowering agents for dyslipidemia were analyzed.

**Results:**

There were 926 HIV-infected patients undertaking ART in the hospital during the study period. Among them, 23.2% of patients undergoing lopinavir-based regimen, 8.4% efavirenz-based regimen, 4.2% darunavir-based regimen, 3.3% nevirapine-based regimen, 2.4% raltegravir-based regimen and 2.3% atazanavir-based regimen developed dyslipidemia. There were 76 patients (8.2%) who needed management of dyslipidemia (Table 1). Among them, 97% received lipid-lowering agents, and 17% switched to lipid-friendly ART (atazanavir, boosted atazanavir, boosted darunavir, nevirapine or raltegravir) despite statins or fibrates used. Mean values (mg/dL) of TC/ TG/LDL were, respectively, 279/422/139 before enrolment, 209/270/114 at 4–12 weeks and 206/250/121 at 48 weeks (*p<*0.05 for baseline compared to 4–12 weeks and 1 year, respectively). No obvious changes in HDL were noted. In Cox proportional hazard model, patients who received lopinavir (adjusted hazard ratio [aHR], 0.293; 95% confidence interval [CI], 0.110–0.784; *p*=0.015) or efavirenz (aHR, 0.185; 95% CI, 0.072–0.447; *p*=0.005) were less likely to achieve normalization of TC (<200 mg/dL) and TG (<200 mg/dL). Modification of ART (aHR, 1.807; 95% CI, 0.828–3.944; *p*=0.137) did not change the outcome (Figure 1).

**Conclusions:**

Efavirenz and lopinavir were independent factors for the persistence of dyslipidemia despite adding lipid-lowering agents. ART associated with a favourable lipid profile would be considered in the modern era, and this certainly leaves the question of cost versus benefit.

**Figure 1:**
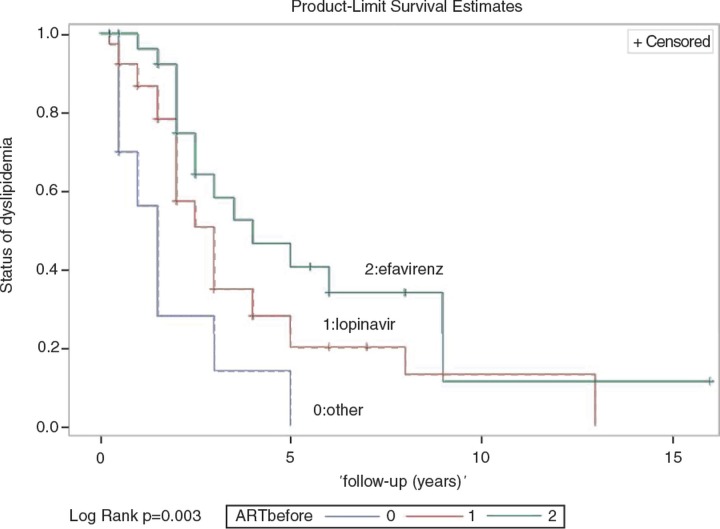
Kaplan-Meier analysis for 76 HIV-infected patients who received ART modification or add-on lipid-lowering agents.

**Table 1:** Characteristics and lipid profile in 76 HIV-infected patients who developed dyslipidemia after ART treatment in Taoyuan, Taiwan

Characteristics	Patients number or mean	Percentage (SD)
Total number	76	100%
Female	11	14.5%
Age	36.1	(8.9)
Body mass index	22.94	(3.79)
HIV transmission category		
Injection drug users	11	14.5%
Heterosexual	13	26.3%
Men who have sex with men	52	68.4%
Nadir CD4 T cell counts	220.9 (median 136)	(249.5)
Nadir HIV viral load (log10)	4.95	(0.92)
Years between HIV diagnosis and regimen modification	3.09	(2.89)
Fibrate added on	16	21.5%
Statin added on	64	84.2%
Switch to lipid-friendly ART after lipid-lowering agents	13	17.1%
Baseline total cholesterol/triglyceride/high-density lipoprotein/low-density lipoprotein (mg/dL)	279/422/45/139	(85/618/15/57)
4–12 weeks total cholesterol/triglyceride/high-density lipoprotein/low-density lipoprotein (mg/dL)	209/270/47/114	(42/292/15/41)
48 weeks total cholesterol/triglyceride/high-density lipoprotein/low-density lipoprotein (mg/dL)	206/250/44/121	(41/205/13/36)

